# The equigenic effect of greenness on the association between education with life expectancy and mortality in 28 large Latin American cities

**DOI:** 10.1016/j.healthplace.2021.102703

**Published:** 2021-11

**Authors:** Mika R. Moran, Usama Bilal, Iryna Dronova, Yang Ju, Nelson Gouveia, Waleska Teixeira Caiaffa, Amélia Augusta de Lima Friche, Kari Moore, J. Jaime Miranda, Daniel A. Rodríguez

**Affiliations:** a Institute for Urban and Regional Development, University of California Berkeley, Berkeley, CA, USA; b Urban Health Collaborative, Drexel Dornsife School of Public Health, Philadelphia, PA, USA; c Department of Landscape Architecture and Environmental Planning, College of Environmental Design, University of California Berkeley, Berkeley, CA, USA; d Departamento de Medicina Preventiva, Faculdade de Medicina da Universidade de São Paulo, São Paulo, Brazil; e Observatório de Saúde Urbana de Belo Horizonte, Faculdade de Medicina da Universidade Federal de Minas Gerais, Belo Horizonte, Brazil; f CRONICAS Center of Excellence in Chronic Diseases, Universidad Peruana Cayetano Heredia, Lima, Peru; g Department of Medicine, School of Medicine, Universidad Peruana Cayetano Heredia, Lima, Peru; h Department of City and Regional Planning and Institute for Transportation Studies, University of California Berkeley, Berkeley, CA, USA; i Department of Environmental Science, Policy, and Management, University of California, Berkeley, CA, USA

**Keywords:** NDVI, Education, Mortality, Latin America, Urban health

## Abstract

**Background:**

Recent studies highlight the equigenic potential of greenspaces by showing narrower socioeconomic health inequalities in greener areas. However, results to date have been inconsistent and derived from high-income countries. We examined whether urban greenness modifies the associations between area-level education, as a proxy for socioeconomic status, and life expectancy and cause-specific mortality in Latin American cities.

**Methods:**

We included 28 large cities, >137 million inhabitants, in nine Latin American countries, comprising 671 sub-city units, for 2012–2016. Socioeconomic status was assessed through a composite index of sub-city level education, and greenness was calculated using the normalized difference vegetation index. We fitted multilevel models with sub-city units nested in cities, with life expectancy or log(mortality) as the outcome.

**Findings:**

We observed a social gradient, with higher levels of education associated with higher life expectancy and lower cause-specific mortality. There was weak evidence supporting the equigenesis hypothesis as greenness differentially modified the association between education and mortality outcomes. We observed an equigenic effect, with doubling magnitudes in the violence-related mortality reduction by education in areas with low greenness compared to medium-high greenness areas among men (16% [95% CI 12%–20%] **vs** 8% [95% CI 4%–11%] per 1 SD increase in area-level education). However, in contradiction to the equigenesis hypothesis, the magnitude in cardiovascular diseases (CVD) mortality reduction by education was stronger in areas with medium-high greenness compared to areas with low greenness (6% [95% CI 4%–7%] **vs** 1% [95% CI -1%–3%] and 5% [95% CI 3%–7%] **vs** 1% [95% CI -1%–3%] per 1 SD increase in area-level education, in women and men, respectively). Similarly, each 1-SD increase in greenness widened the educational inequality in life expectancy by 0.15 years and 0.20 years, in women and men, respectively. The equigenic effect was not observed in violence-related mortality among women and in mortality due to communicable diseases, maternal, neonatal and nutritional conditions (CMNN).

**Interpretation:**

Our results confirm socioeconomic health inequalities in Latin American cities and show that the equigenic properties of greenspace vary by health outcome. Although mixed, our findings suggest that future greening policies should account for local social and economic conditions to ensure that greenspaces provide health benefits for all, and do not further exacerbate existing health inequalities in the region.

**Funding:**

Wellcome Trust (Grant, 205177/Z/16/Z).

## Highlights

• This study is the first to examine the equigenesis hypothesis of greenspace in Latin America.
• Higher education was associated with higher life-expectancy and lower cause-specific mortality.
• Education disparities in violence-related mortality were narrower in greener areas.
• Greener areas had wider education disparities in life-expectancy and CVD mortality.

## 1 Introduction

Accumulating evidence worldwide (Rahimi-Ardabili et al., 2021; Rigolon et al., 2018; Markevych et al., 2017) suggests that greenspace contributes to a wide range of health outcomes through three distinct yet overlapping pathways: *mitigation* - reducing environmental exposure (e.g., air pollution, heat, noise); *recreation* – providing space for activity and socialization; and *restoration* – reducing stress and aggression and improving well-being (Markevych et al., 2017). Yet, in urban regions greenspaces are often unequally distributed in a way that disproportionally benefits affluent groups (Rigolon et al., 2018; Hoffimann et al., 2017; Wolch et al., 2014; Song et al., 2021). Other evidence also exists linking deprivation with higher availability of greenspaces but of lower quality (Mears et al., 2019; Wang et al., 2021a), which might ultimately outweigh their health benefits for local population. Such inequalities in greenspace have been further linked to socioeconomic disparities in health outcomes, such as obesity, cardiovascular disease and well-being (Gordon-Larsen et al., 2006; Jennings and Gaither, 2015). Greenspaces, especially those free and accessible, may have health effects that are especially beneficial for socioeconomically disadvantaged groups, who usually have lower access to affordable alternative health-promoting resources. This potential of greenspace to mitigate health inequalities was articulated by Mitchel and Popham as the “equigenesis hypothesis of greenspace” (Mitchell, 2013; Mitchell and Popham, 2008) hereafter the “equigenesis hypothesis”, suggesting that, while different places may experience similar socioeconomic gaps, the extent to which these translate into health inequalities is weakened in greener areas (Mitchell, 2013). Despite its intuitive appeal, the equigenesis hypothesis has only recently attracted research interest. Although research on greenspace and health is rapidly growing worldwide (Rahimi-Ardabili et al., 2021; Rigolon et al., 2018), studies that articulate and examine the equigenesis hypothesis are still relatively scarce, and mostly from high income countries, with some research results supporting the equigenesis hypothesis (Mitchell and Popham, 2008; Brown et al., 2018; Mitchell et al., 2015), while others not (Feng and Astell-Burt, 2017; Sugiyama et al., 2016).

Green space's equigenic effects are likely to occur by providing greater health benefits to deprived compared to affluent individuals and populations (Mitchell et al., 2018) (also referred to as “leveling up” (Mitchell, 2013)). For example, deprived individuals may use greenspaces more because of the lack of other affordable opportunities for recreation (Mitchell, 2013; Wang et al., 2021b). Alternatively, equigenesis may also occur by posing greater health risks among affluent compared to economically disadvantaged individuals and populations (also referred to as “leveling down” (Mitchell, 2013)). However, this is less likely to be the case for greenspaces, which are more likely to offer health benefits than risks, and when introducing risks, these would more likely affect deprived populations, for example, when located in underserved areas, park might be prone to attract crime and social disorder (Groff and McCord, 2012).

Cities in Latin America suffer from significant deficits of greenspaces, especially in low income areas (Rigolon et al., 2018). Yet, despite their scarcity, greenspace in Latin American cities provide multiple health benefits (Amorim et al., 2010; Jáuregui et al., 2016). This study explores the equigenesis hypothesis in urban Latin America through the following question: Does greenness modify the associations between area-level education, as a proxy for socioeconomic status, with life expectancy and cause-specific mortality? Based on existing evidence (Brown et al., 2018; Mitchell et al., 2015; Dadvand et al., 2012; McEachan et al., 2016), we hypothesize that education inequalities in life expectancy and cause-specific mortality are narrower in greener areas.

## 2 Methods

### 2.1 Study setting

Data came from the SALURBAL (“Salud Urbana en America Latina” or Urban Health in Latin America) study, which includes a harmonized database of health, social and built-environment variables for all cities above 100,000 residents in eleven Latin American countries (Quistberg et al., 2019). The current analysis is focused on a SALURBAL sub-sample of 28 large cities (Appendix 1) in nine Latin American countries (Argentina, Brazil, Chile, Colombia, Mexico, Panama and El Salvador) for the period 2012–16. A total of 671 sub-city units was included in our sample (>10 per city). Sub-city units refer to *comunas*, *municipios* or *distritos* depending on the country (Quistberg et al., 2019) (Appendix 2).

### 2.2 Variables

This analysis includes four mortality outcomes, one main exposure (education), and one effect modifier (greenness) at the sub-city level, as well as several covariates at the city- and sub-city levels (Appendix 2).

#### 2.2.1 Outcomes

The four outcomes of this study were life-expectancy at birth and three categories of cause-specific mortality that correspond with three main pathways through which greenspaces benefit health (Markevych et al., 2017): (1) *Communicable, maternal, perinatal and nutritional conditions (CMNN)*, which may reflect the mitigation pathway; (2) *Cardiovascular diseases (CVD)*, which may reflect the *recreation* mechanism; and (3) *Violence-related mortality*, which may reflect the *restoration* mechanism. As aforementioned, these pathways may overlap. For example, greenspace may reduce CVD mortality by both increasing activity (*recreation*) and reducing stress (*restoration*). On the other hand, greenspaces’ mitigation effects, for example by reducing heat, are not limited to reducing the risk for CMNN diseases but may also reduce risks for CVD (Gasparrini et al., 2012) and violence-related mortality (homicides) (Ceccato, 2005; Trujillo and Howley, 2019). We use these three pathways as a simplified framework to guide our analysis, while acknowledging these nuances and accounting for such potential overlaps when interpreting the results.

Life expectancy is an aggregate measure of mortality at all ages accounting for the age-structure of the population. Specifically, life expectancy at birth represents the average number of years a person born today is expected to live if current age-specific mortality rates hold constant over time. We obtained mortality data from vital registration systems and population denominators from statistics offices in each country, for the period 2012–2016 (except for El Salvador, where we used data for the period 2010–2014 due to restricted data availability). We addressed three key challenges of vital registration data: (1) we imputed missing age and sex in <0.1% records; (2) we redistributed ill-defined causes of death proportionally by age, sex, country and year, in 3% of records (Bilal et al., 2021); and (3) we addressed in complete coverage of mortality by using an ensemble of death distribution methods at the city-level (Bilal et al., 2021).

We estimated all-cause (for life expectancy) and cause-specific mortality rates using a Bayesian model that acknowledges uncertainty in the calculation of the coverage of mortality counts, based on the model by Schmertmann and Gonzaga (2018). To properly acknowledge uncertainty in the estimation of mortality rates, we sampled 1000 age-sex (all-cause and cause-specific) mortality rates for every sub-city from the posterior distributions resulting from the model. We computed life expectancy using life tables, and computed age-adjusted CMNN, CVD, and violence mortality rates using direct standardization, with the WHO 2000–2025 World Population as the referent population. More details on these three challenges of vital registration and the methods we implemented to address them are available elsewhere (Bilal et al., 2021). For descriptive purposes, we used the median (from the 1000 posterior estimates) life expectancy and age-adjusted CMNN, CVD and violence mortality rates.

#### 2.2.2 Main exposure: Education

We measured area-level socioeconomic status using attained education at the sub-city level. Specifically, we computed a composite index using the percent of people aged 25 years or older with completed secondary education and the percent of people aged 25 years or older with completed university education. We calculated z-scores for each variable and summed together to get the composite index. Data on education were obtained from the most recent national censuses and harmonized to allow for international comparability (see Appendix 2) (Quistberg et al., 2019).

#### 2.2.3 Effect modifier: Greenness

Greenness was derived from satellite images (product MOD13Q1.006 from Moderate-Resolution Imaging Spectroradiometer (MODIS) Terra sensor) at a temporal resolution of 16 days and spatial resolution of 250 m (Didan, 2015). The Normalized Difference Vegetation Index (NDVI) was used as an indicator of vegetation greenness with values ranging from −1 to +1, where higher values reflect higher coverage or biomass of green vegetation. NDVI is a common proxy for greenspaces used in prior studies on greenspace and health (Markevych et al., 2017) and on the equigenesis hypothesis (Brown et al., 2018; McEachan et al., 2016; Crouse et al., 2017). The annual maximum NDVI value per 250 m grid cell was identified. Then, the median value of annual maximum NDVIs was identified within each area unit (city/sub-city) for each year between 2012 and 2016 (overlapping the years of mortality data). Finally, the median values between 2012 and 2016 were averaged to get a 5-year aggregate measure of greenness.

#### 2.2.4 Covariates

All of the models were adjusted for covariates at the city and sub-city levels. Covariates at the sub-city level included Euclidean distance between the sub-city geographic centroid to the city center, and the proportion of sub-city population aged 0–15 years and 65 years or older. The Euclidean distance between the sub-city geographic centroid to the city center was included because our data showed greater variability in greenness near city centers, and generally higher greenness as distance from the center increases (data not reported). We adjusted for sub-city level age distribution to account for potential differences in the age structure within each city. At the city level, covariates included the proportion of the total area that is built-up (Esch et al., 2018; FRAGSTATS, 2021), total population, climate zone (temperate, tropical, arid) (Rubel and Kottek, 2010) and greenness (Didan, 2015). The proportion of the total area that is built-up was used to adjust for variations in cities’ administrative area. City-level greenness was added to account for its macro scale environmental impact and for differences in greenness between cities, as opposed to our main interest, i.e., differences in greenness within cities. We also controlled for climate zone because the quality of greenspaces and therefore some of their effects on health may vary across different climate zones.

### 2.3 Analysis

We first described the distribution of study variables. For this, we classified all sub-cities and cities as above or below the median in greenness and education, and created four categories: low greenness/low education, low greenness/high education, high greenness/low education, and high greenness/high education.

To examine whether the associations between educational inequalities in life expectancy and log(mortality) varied by sub-city greenness, we used multilevel linear models of sub-cities nested within cities, with country fixed effects. For each outcome and sex, we fitted a model including sub-city greenness, socioeconomic status (education) and an interaction term between sub-city greenness and socioeconomic status (education). Both sub-city greenness and education were centered at their mean and scaled by their standard deviation (SD).

Coefficients for the life expectancy models are interpreted as changes in life expectancy (in years) per 1-SD change in either area-level education or greenness, whereas their interaction coefficients are the change in the educational inequality in life expectancy (the statistical effect of education on life expectancy, henceforth – social gradient) per 1-SD change in greenness. Exponentiated coefficients for the cause-specific mortality models are interpreted as the relative percent change in cause-specific mortality per 1-SD change in either area-level education or greenness, whilst their exponentiated interaction coefficients are the relative rate ratios (RRR), or the change in the educational inequality in cause-specific mortality (i.e., the social gradient as quantified by the statistical effect of education on cause-specific mortality) per 1-SD change in greenness. Since all educational inequalities were found to be significant and in the hypothesized direction (higher education associated with higher life expectancy and lower cause-specific mortality), we interpreted interaction coefficients as narrowing inequalities if they made the statistical effect of education closer to the null, and as widening inequalities if they made the statistical effect of education further away from the null.

All analyses were conducted in R 4.0.2, while the Bayesian models were implemented in JAGS v4. Estimates and 95% confidence intervals (95% CIs) presented in figures were obtained from a linear combination of the main education coefficient and the interaction coefficient, using the glht function of the multcomp package in R v4.0.0. Last, we checked model diagnostics by: (a) examining the normality of both sub-city and city-level residuals through the use of histograms, and (b) homoscedasticity of sub-city level residuals by plotting residual vs fit, all separately for each outcome and gender.

## 3 Results

Table 1 presents the median and interquartile range of the variables in the total sample and in the four sub-samples defined by different combinations of high/low education and greenness (based on median splits) at the sub-city and city levels. We included a total of 671 sub-city units in 28 cities with a median population of 3 million people. The median life expectancy was 78·8 for women and 73·5 for men. The most common mortality cause was CVD (274·6 and 400·0 per 100,000 in women and men respectively), followed by CMNN (52·3 and 76·6 per 100,000 in women and men respectively) and violence-related deaths (4·7 and 28·5 per 100,000 in women and men respectively). The median percentage of population per sub-city with high-school or university education was 35·4%, of which 26·2% had high-school education and 9·2% had university education. The median NDVI (greenness) value was 0·7 and 0·6 at the sub-city and city levels, respectively. These values represented a high green vegetation coverage, reflecting that most of our sample cities are located in temperate and tropical climate zones (13 and 10 of the 28 cities, respectively).

**Table 1:** Summary statistics of study variables (median, IQR) in the total sample and by sub-city and city area-level education and greenness.

			Total sample	Low Education		High Education	
				Low Greenness	High Greenness	Low Greenness	High Greenness
Number of sub-city units^a^			671	119	217	217	118
Sub-city life expectancy and mortality							
Life expectancy		Women	78·8 [77·5; 80·7]	78·1 [77·1; 80·1]	78·2 [77·5; 79·1]	79·6 [77·9; 81·6]	80·3 [78·6; 81·3]
		Men	73·5 [71·6; 76·1]	73·1 [71·6; 74·7]	72·3 [70·9; 74·1]	76·0 [72·2; 78·3]	75·0 [72·7; 76·8]
CMNN mortality		Women	52·3 [41·8; 78·9]	44·7 [37·5; 49·8]	66·9 [49·1; 79·1]	81·4 [61·1; 99·7]	46·8 [32·7; 75·2]
		Men	76·6 [59·3; 111·2]	61·4 [52·0; 68·9]	93·1 [68·9; 116·9]	111·2 [81·8; 130·0]	75·4 [54·2; 100·1]
CVD mortality		Women	274·6 [224·3; 339·4]	343·6 [251·1; 376·5]	297·6 [262·2; 334·1]	234·0 [161·1; 298·4]	243·2 [222·0; 274·6]
		Men	400·0 [309·7; 469·7]	446·4 [367·3; 512·8]	444·4 [383·6; 488·9]	303·5 [209·9; 436·3]	332·6 [297·5; 391·6]
Violence-related mortality		Women	4·7 [3·5; 6·5]	5·5 [4·5; 6·6]	4·9 [3·6; 8·3]	4·2 [0·8; 6·1]	3·9 [3·3; 5·1]
		Men	28·5 [18·5; 43·7]	31·5 [23·7; 40·6]	25·3 [18·4; 78·7]	16·9 [4·0; 36·7]	32·9 [22·0; 49·7]
Sub-city level characteristics							
Greenness (sub-city level)			0·7 [0·5; 0·8]	0·7 [0·5; 0·8]	0·8 [0·6; 0·8]	0·5 [0·2; 0·7]	0·8 [0·7; 0·9]
High school education (%)			26·2 [19·7; 33·3]	21·3 [16·0; 27·9]	23·4 [17·0; 29·2]	39·5 [26·3; 50·7]	27·5 [22·0; 32·4]
College education or higher (%)			9·2 [5·5; 16·9]	6·5 [3·6; 10·7]	7·2 [4·1; 11·8]	13·0 [8·8; 20·0]	14·0 [8·7; 22·4]
% population aged 0-15			7·9 [7·0; 8·6]	7·9 [6·9; 8·8]	8·6 [7·0; 9·0]	8·2 [7·1; 8·4]	8·4 [7·0; 8·4]
% population aged 65+			7·1 [6·8; 8·1]	6·9 [5·9; 8·0]	8·1 [6·8; 11·2]	7·1 [7·0; 7·4]	7·5 [6·8; 8·1]
Distance to city center (km.)			19·0 [9·9; 31·4]	23·4 [13·5; 34·2]	17·6 [9·6; 33·1]	14·6 [7·8; 26·9]	18·8 [11·0; 27·8]
City level characteristics							
Number of cities^b^			28	6	8	8	6
Greenness (city level)			0·6 [0·5; 0·6]	0·5 [0·4; 0·5]	0·6 [0·6; 0·6]	0·5 [0·2; 0·5]	0.6 [0.6; 0.7]
Total population (millions)			3·0 [0·8; 5·2]	4·6 [3·3; 5·9]	2·8 [1·5; 3·7]	1·9 [0·6; 8·7]	2.1 [0.6; 2.8]
Population growth (%/5 years)			4·5 [3·3; 5·8]	3·4 [3·0; 5·5]	4·9 [3·0; 5·9]	4·5 [3·9; 4·9]	5.1 [4.1; 6.5]
% built-up			13·0 [8·0; 18·3]	16·0 [10·7; 22·4]	13·0 [11·8; 16·7]	12·7 [6·0; 17·8]	9.0 [5.3; 15.9]
Climate zone group - N [%]	Arid		5 [18%]	2 [33%]	0 [0%]	3 [38%]	0 [0%]
	Tropic		10 [36%]	1 [17%]	3 [37%]	1 [12%]	5 [83%]
	Temperate		13 [46%]	3 [50%]	5 [63%]	4 [50%]	1 [17%]

a Selected sub-city characteristics and life expectancy/mortality were calculated in the total sample and in four categories defined by high/low sub-city greenness and education using sub-city median values as cut-offs/thresholds (e.g., 119 sub-cities had greenness and education values that were below the median in all sub-cities were thus defined as low greenness/low education).

b Selected city-level characteristics were calculated in the total sample and in four categories defined by high/low city greenness and education values using city median values as cut-offs/thresholds (e.g., 6 cities had greenness and education values below the median in all cities and were thus defined as low greenness/low education).

To examine the equigenesis hypothesis, greenness and education were used in their continuous form (Table 2). Table 2 reports adjusted models estimating life expectancy and cause-specific mortality by sub-city education and the interaction by greenness, adjusted for covariates. Education was consistently associated with higher life expectancy and lower cause-specific mortality in both sexes. For women, in sub-cities with average greenness, every additional SD of education is associated with 0·34 years increase in life expectancy, with a 4·1% reduction in CMNN and CVD, and a 4·0% reduction in violence-related mortality, respectively. This association in men was stronger for two of the outcomes, i.e. every additional SD of education is associated with 0·51 years increase in life expectancy, and with a 10·5% reduction in violence-related mortality, respectively. For informative purposes, we also estimated the association between greenness and the outcomes of interest (Table 2), showing no association between greenness with life expectancy and mortality in women, while in men, in sub-cities with average education, every additional SD of greenness is associated with 0·39 years higher life expectancy and with a 5% reduction in CMNN mortality.

**Table 2:** Associations between area-level education and life expectancy and cause-specific mortality, and interaction effects by sub-city greenness (n = 671).

	Life expectancy	CMNN mortality	CVD mortality	Violence-related mortality
Association by sex	β (95% CI)*	RR (95% CI)^	RR (95% CI)^	RR (95% CI)^
Women				
Education	**0·34 (0·23; 0·45)**	**0·96 (0·94; 0·97)**	**0·96 (0·95; 0·97)**	**0·96 (0·93; 0·99)**
Greenness	−0·04 (−0·33; 0·24)	**0·96 (0·93; 1·00)**	1·01 (0·98; 1·04)	0·99 (0·92; 1·06)
				
Men				
Education	**0·51 (0·36; 0·65)**	**0·96 (0·94; 0·97)**	**0·96 (0·95; 0·98)**	**0·90 (0·87; 0·92)**
Greenness	**0·39 (0·03; 0·75)**	**0·95 (0·91; 0·99)**	0·99 (0·96; 1·02)	**0·93 (0·87; 1·00)**
				
Interaction Education*Greenness	β (95% CI)*	RRR (95% CI)^	RRR (95% CI)^	RRR (95% CI)^
				
Women	**0·15 (0·05; 0·24)**	0·99 (0·98; 1·00)	**0·99 (0·98; 0·99)**	1·01 (0·98; 1·03)
Men	**0·20 (0·08; 0·32)**	0·99 (0·98; 1·00)	**0·99 (0·98; 1·00)**	**1·03 (1·01; 1·06)**

Footnote.

* Results for life expectancy are beta coefficients (95% CI), interpreted as the increase in years of life expectancy per 1 SD increase in education or greenness, or their interaction for the Education*Greenness coefficient.

^ Results for cause-specific mortality are Rate Ratios (95% CI), interpreted as the relative change in CVD/CMNN/Violence-related mortality per 1 SD increase in education or greenness, or Relative Rate Ratios (95% CI) for the Education*Greeness interaction.

E.g. in men, a 1 SD increase in education is associated with 0·51 years higher life expectancy. The interaction term for life expectancy shows that for each 1-SD increase in greenness, the educational inequality becomes wider by 0.15 and 0.20 years for women and men, respectively. For violence-related mortality in men, a 1-SD increase in education is associated with a 10% (0·90–1 = −0·10 or −10%) reduction in violence-related mortality, and the interaction term shows that for each 1-SD increase in greenness, the educational inequality is reduced by 3% (1.03–1 = 0.03 or 3%). Specifically, the association between education and violence-related mortality in areas with +1-SD greenness would be 0.90 x (1.03 x +1) = 0.93, meaning that in these areas a 1-SD increase in education is associated with a 7% (0.93–1 = −0.07, or −7%) reduction in violence-related mortality in men.

For women, in the case of CVD mortality, a 1-SD increase in education is associated with a 4% (0.96–1 = −0.04, or −4%) decrease in CVD mortality, and the interaction term shows that for each 1-SD increase in greenness, the educational inequality is increased by 1%. Specifically, the association between education and CVD mortality in areas with +1-SD greenness would be 0.96 x (0.99 x +1) = 0.95, meaning that in these areas a 1-SD increase in education is associated with a 5% (0.95–1 = −0.05, or −5%) reduction in CVD mortality in women.

All dependent variables are in Z-score units. Coefficients with a p-value <0·05 are in bold.

There was evidence of a modification by greenness on the association between education and life expectancy (both sexes), CVD mortality (in women) and violence-related mortality (in men). We found that each 1-SD increase in greenness widened the educational inequality in life expectancy by 0.15 years and 0.20 years, in women and men, respectively. A similar pattern of widening educational inequalities was observed for CMNN and CVD mortality, where a 1-SD increase in greenness widened the educational inequality in CMNN and CVD mortality by 1%, although this was only significant for CVD mortality in women. On the contrary, a 1-SD increase in greenness narrowed the educational inequality in violence-related mortality by 1% and 3%, in women and men, respectively (Table 2).

**Fig. 1:**
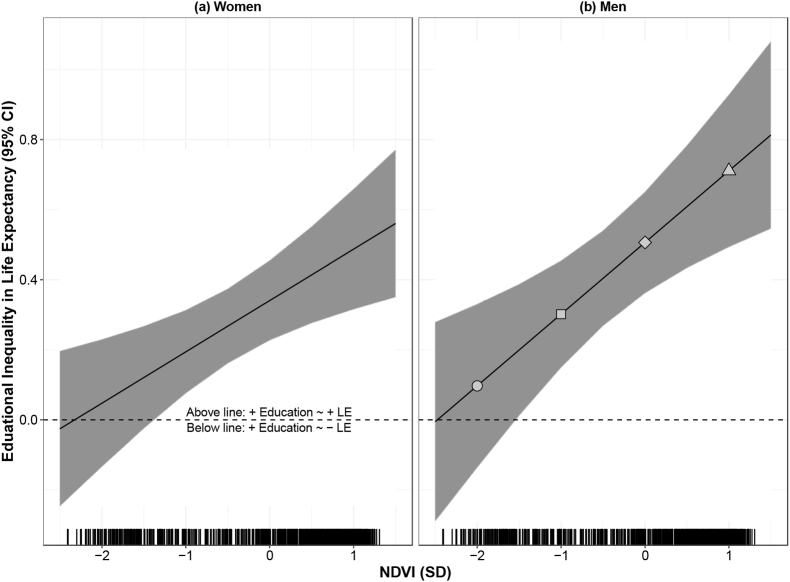
Change in life expectancy in women (a) and men (b) per 1-SD increase in education as a function of greenness. Footnote: Effect estimates were derived from Table 2, including coefficients for education, greenness, their interaction, and all confounders. Estimates and 95% CIs obtained from a linear combination of the main education coefficient and the interaction coefficient, using the glht package in R. The X-axis refers to greenness (as per NDVI), the Y-axis is the educational inequality (or social gradient). The lines (bands) represent the coefficients (95% CIs) of the association between education and life expectancy, by levels of greenness. The horizontal dashed line represents a lack of association between education and life expectancy. To ease interpretation, when the education coefficient is closer to the null (horizontal dashed line), the educational inequality (or social gradient by education) is narrower. For example, Fig. 1 b. Includes four points of reference along at −2, −1, 0, and +1 SD of greenness. A 1-SD increase in education is associated with: a 0·10-year (95% CI -0·14 to 0·33) increase in life expectancy for men in areas with low greenness (-2SD, circle); a 0·30-year (95% CI 0·16 to 0.0·43) increase in life expectancy for men in areas with medium-low greenness (−1 SD, square); a 0·51-year (95% CI 0·36 to 0·65) increase in life expectancy for men in areas with average greenness (0SD, rhombus); and a 0.71-year (95% CI 0·49 to 0·93) increase in life expectancy for men in cities with medium-high greenness (+1SD, triangle), and shows that the educational inequality (social gradient by education) is wider in the greener areas (+1SD, triangle). Estimates along these four points of references (−2, −1, o and +1 SD of greenness) for graphs 1(a-b) are also presented in Appendix 3a.

**Fig. 2:**
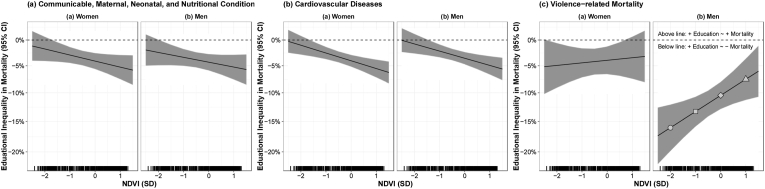
Relative change in cause-specific mortality (communicable, maternal neonatal and nutritional conditions (a); cardiovascular diseases (b); and violence-related mortality (c)) per 1-SD higher in education as a function of greenness. Footnote: Effect estimates were derived from Table 2, including coefficients for education, greenness, their interaction, and all confounders. Estimates and 95% CIs obtained from a linear combination of the main education coefficient and the interaction coefficient, using the glht package in R. The X-axis refers to greenness (as per NDVI), the Y-axis is the educational inequality (or social gradient). The lines (bands) represent the coefficients (95% CIs) of the association between education and cause-specific mortality, by levels of greenness. The horizontal dashed line represents a lack of association between education and cause-specific mortality. To ease interpretation, when the education coefficient is closer to the null (horizontal dashed line), the educational inequality (or social gradient by education) is narrower. For example, Fig. 2 c.b. Includes four points of reference along at −2, −1, 0, and +1 SD of greenness. A 1-SD increase in education is associated with: a 16% (RR = 0.84, 95% CI 0·80 to 0·88) reduction in violence-related mortality in men in areas with low greenness (-2SD, circle); a 13% (RR = 0.87, 95% CI 0·84 to 0·89) reduction in violence-related mortality in men in areas with medium-low greenness (−1 SD, square); a 10% (RR = 0.90, 95% CI 0·87 to 0·92) reduction in life expectancy for men in areas with average greenness (0SD, rhombus); and a 8% (RR = 0.92, 95% CI 0·89 to 0·96) reduction in violence-related mortality in men in areas with medium-high greenness (+1SD, triangle), and shows that the educational inequality (social gradient by education) is narrower in the greener areas (+1SD, triangle). Estimates along these four points of references (−2, −1, o and +1 SD of greenness) are also presented in Appendix 3a.

Fig. 1 and Fig. 2 present differences in the educational inequality in life expectancy and cause-specific mortality associated by sub-city greenness, and detailed estimates are provided in Appendix 3. In terms of life expectancy, in both women and men, we found that higher area-level education was associated with higher life expectancy across levels of greenness, but this association was stronger in greener areas, meaning that, against the equigenesis hypothesis, the educational inequalities in life expectancy were wider in greener areas (Fig. 1). Similarly, we found that higher area-level education was associated with lower CMNN and CVD mortality across levels of greenness in women and men and, also against the equigenesis hypothesis, educational inequalities in mortality were wider in greener areas (Fig. 2).

Model diagnostics revealed no departure from normality for both sub-city and city-level residuals, and equal variances of sub-city level residuals across levels of the outcome.

## 4 Discussion

In this multi-country study of 671 sub-city areas in 28 large cities with a total of over 137 million inhabitants, we confirmed the existence of a social gradient, so that life expectancy was higher, and cause-specific mortality was lower, in areas with higher-educational attainment. However, we also found that greenness modified this association, specifically for life expectancy (both sexes), CVD mortality (in women), and violence-related mortality (in men). For the equigenesis hypothesis, we found that the social gradient in violence-related mortality was narrower in greener areas, especially in men. In contrast with the equigenesis hypothesis, we found wider inequalities in life expectancy and in CVD mortality in greener areas. The equigenesis hypothesis is therefore confirmed by our results for violence-related mortality but challenged by our results for life expectancy and CVD mortality.

We found that greener areas had narrower educational disparities in violence-related mortality, especially in men, as the educational inequalities in violence-related mortality were twice as strong in low greenness areas as compared to medium-high greenness areas (16% vs 8%). These findings, at least in terms of their direction, albeit for different outcomes, are in line with other studies from high-income countries. For example, Mitchell and Popham (2008) found that, in a national sample of over 40 million UK residents, inequalities in all-cause and in circulatory system disease mortality were narrower in populations living in greener areas. A few other studies have similarly shown associations between higher greenspace availability and reduced socioeconomic inequalities in various health outcomes, including birth weight (Dadvand et al., 2012), Alzheimer and depression (Brown et al., 2018), mental well-being (Mitchell et al., 2015), and women's depression during pregnancy (McEachan et al., 2016).

The narrower inequalities in men's violence-related death that were observed in greener areas in our study can be explained by greenspaces' restoration effect, as exposure to nature by seeing and sensing it as well as simply having it nearby, can reduce stress, anger, and aggression, which can then, ultimately, reduce violence (Branas et al., 2018; Kuo and Sullivan, 2001). Greenspaces can also indirectly reduce violence by providing safe spaces for activity and social interactions, which contribute to community social capital (Bedimo-Rung et al., 2005). From a planetary health perspective, the stronger protective effect of greenness from men's violence mortality in areas with low education (Appendix 3b) could be attributed to urban heat abatements, as greenspace were found to reduce spikes in violent behaviors that were attributed to extreme heat (Ceccato, 2005; Trujillo and Howley, 2019), especially in low-income urban areas where heat-violent crime associations were shown to be stronger (Heilmann and Kahn, 2019). Overall, our results suggest that in economically disadvantaged neighborhoods, these roles of greenspace in restoration, recreation and mitigation become more essential and even critical given that other alternatives are likely to be unavailable and/or unaffordable for residents (Moore et al., 2008).

While theoretically appealing, these explanations have some caveats that merit attention. Inasmuch as greenspaces can improve community safety, when located in high poverty communities, greenspaces can also provide hidden and isolated spaces away from formal surveillance and enforcement (Rigolon, 2016a). Alongside this, a recent study from Latin America (Moran et al., 2020) suggests that reported neighborhood social disorder does not hinder park use and further links reported indigence or begging with increased park use. These findings, in addition to the overall lower quality and maintenance of greenspaces in economically disadvantaged urban areas (Markevych et al., 2017; Bedimo-Rung et al., 2005), challenge our ability to use the restoration mechanism to explain the greater protective effect of greenness from men's violence-related mortality that was observed in low-educated areas (Appendix 3b). To better understand this potential dual nature and of green spaces, future research should account for the socioeconomic context in which greenspaces are located, and use more detailed metrics of greenspaces that are pertinent to understand their associations with violence and crime, such as the spatial configuration and accessibility of greenspaces (Wang and Tassinary, 2019) and the facilities and programs available.

We also found evidence against the equigenesis hypothesis for life expectancy and for CVD mortality. These findings contradict the aforementioned UK study (Mitchell and Popham, 2008), which found narrower CVD mortality gradients in greener areas, but are in line with a recent longitudinal study that followed 1·3 million residents from 30 cities in Canada for 11 years (2001–2011) and similarly showed that the protective effect of greenspace was stronger in more affluent groups (Crouse et al., 2017). Our results could be attributed to green gentrification – the process in which urban greening is accompanied with increases in housing value thereby increasing the financial burden on low-income residents, who eventually might be displaced. This accumulating stress among low-income residents can outweigh the benefit of the new greenspace, and can further ultimately harm their health (Anguelovski et al., 2019), while affluent residents are likely to remain uninfluenced and able to enjoy the recreational benefits offered by the new greenspace (Cole et al., 2019). In other words, our results showing wider inequalities in life expectancy and CVD mortality in greener areas may be traced back to greening policies that further widened existing local socioeconomic gaps, which, in turn, increased health inequalities. While intriguing, this explanation is speculative as our data include greenness availability but lack the historical and socio-economic context of preceding greening policies. To explore the role of green gentrification in shaping the equigenic properties of greenspaces over time, longitudinal research designs are needed, as priorly suggested (Mitchell et al., 2018). In addition, future research should apply a broader and critical perspective by engaging local populations in the research process, and investigating historical, political and socioeconomic contexts in which greenspaces are developed.

Our findings also imply that greenspaces’ protective effect on LE and CVD mortality is stronger in areas with higher education (Appendix 3b). This can be attributed to better quality of greenspaces in wealthier and more educated areas, which may provide more health benefits of various kinds (Rigolon, 2016b). Specifically, greenspaces in wealthier areas are likely to include parks, tree foliage, and recreational areas, whereas in lower income areas greenspaces are more likely to be unmanaged and have lower recreational value (Moore et al., 2008). In addition, wealthier and educated individuals are likely to have more leisure time and to engage in routine physical activity and thus they are more likely to visit greenspaces frequently and enjoy their recreational health benefits (Mitáš et al., 2019).

The use of greenness as a proxy for greenspace is one of several limitations in our study. The lack of a more refined measure of urban greenspace, for example, one that distinguishes between urban parks, urban forests, agricultural land, and the like makes it challenging for interpretation. While this was partially accounted for by adjustment for covariates (e.g., distance to city center), future research can benefit from using more nuanced greenspace metrics accounting for the number of greenspaces available, their spatial configuration and accessibility, as well as separating more clearly recreational spaces, such as parks, from other urban elements contributing to greenness, such as street tree cover. In addition, the use of education as a single proxy indicator for deprivation might fail to capture other deprivation aspects, such as income, living conditions, etc. However, this limitation might be less severe given prior studies from the same databased as ours that showed consistent associations between education and mortality in Latin American cities (Bilal et al., 2021; Bilal et al., 2019). Moreover, we use education as an aggregate measure based on data from different years for different countries, depending on the year of the census. However, as shown in a previous study, while absolute values of education levels may change, the rank between sub-cities tends to be conserved over time (Bilal et al., 2019). Regarding our outcomes, we acknowledge that the CMNN category is broad, although it includes causes with similar etiologies, and further disaggregation is complicated by the low CMNN mortality rates in our cities (Bilal et al., 2021). Moreover, while we corrected for three key issues in vital registration data (missing data, ill-defined causes of death, and under-registration of deaths), there is the possibility that these corrections have been insufficient. In addition, the ecological design of the study makes it difficult to ascertain whether individuals in greener areas indeed have more access to greenspaces and use them more. This is further amplified by the relatively large sub-city units that were used in our analysis to allow for international comparability, but at the same time may incur a smoothing effect as variations may be greater within unites than between units. Finally, as a cross-sectional analysis, our study allows the estimation of associations but not causality. It is possible that selective migration results in higher-income residents living near greenspaces with the highest recreational value and low-income residents with limited access to these areas.

Despite these limitations, our study has several merits that are noteworthy. To our knowledge, this study is pioneering the exploration of the equigenesis hypothesis of greenspaces in Latin America, a vulnerable region due to its high urbanization rate, high intra-urban inequalities, and a deficit and unequal distribution of urban greenspace (Rigolon et al., 2018). The breadth and diversity of the study sample, including 28 cities in nine countries representing three different climate zones, is another merit of this study as, to our knowledge, only one prior study on the equigenesis hypothesis used an international sample (Mitchell et al., 2015), while others mostly focused on one country (Mitchell and Popham, 2008; Feng and Astell-Burt, 2017) or single cities (Brown et al., 2018; Dadvand et al., 2012). Lastly, the use of mortality outcomes that correspond with the different health benefits provided by greenspaces (Markevych et al., 2017) facilitated the results interpretation.

In summary, we examined the equigenesis hypothesis of greenspaces in 28 large Latin American cities. We found that higher area-level education is associated with higher life expectancy and lower mortality rates, and that these associations vary by level of greenness. In support of the equigenesis hypothesis, greener areas showed narrower education inequalities in violence-related mortality, but, against the equigenesis hypothesis, we found wider education inequalities in life expectancy and CVD mortality in greener areas. Whether the associations of education with mortality outcomes are weaker (as in violence-related mortality) or stronger (as in life expectancy and CVD mortality) in greener areas, these results point at investing in high-quality greenspace that are accessible to all as a promising strategy to improve health and reduce existing intra-urban inequalities in life expectancy and mortality in Latin American cities. Although mixed, our findings indicate that greenspaces may play a role in promoting urban health and in shaping intra-urban health inequalities. Given the already wide intra-urban inequalities in Latin American cities, and the sparse and unequal distribution of urban green spaces, greening policies need to make a concerted effort to ensure that unequal access to green spaces does not exacerbate existing health inequalities. This could be achieved by engaging local populations in the planning process and accounting for the historical, socioeconomic and cultural characteristics and dynamics of local environments.

## Research in context

### Evidence before this study

Greenspaces provide a wide range of planetary and human health benefits. In recent years, the potential of greenspaces to reduce health inequalities, often referred to as “the equigenesis hypothesis of greenspaces”, has gained increased research interest. However, results to date are not entirely consistent and arise mostly from high-income countries.

### Added value of the study

Our research articulates and tests the equigenesis hypothesis in Latin America, a vulnerable region with a high urbanization rate, wide intra-urban inequalities, and a deficit and unequal distribution of greenspaces in cities. Our analysis included 671 sub-cities in 28 cities from nine countries in the region. Our results confirm the well-described associations between education and mortality outcomes, and further suggest that greenness differentially modified these associations in opposite directions for different outcomes. In support of the equigenesis hypothesis, greener areas showed narrower social gradient for violence-related mortality in men. However, greener areas also showed wider social gradients for life expectancy and cardiovascular diseases (CVD) mortality, thereby contradicting the equigenesis hypothesis. The equigenic effect was not observed nor contradicted in violence-related mortality among women and in mortality due to communicable diseases, maternal, neonatal and nutritional conditions (CMNN).

### Implications of available evidence

Our findings demonstrate the important role of green spaces in promoting public health and in shaping existing urban health inequalities. Our findings partly confirm and partly reject the equigenesis hypothesis by linking higher greenness with narrower education inequalities in violence-related mortality, but with wider education inequalities in life-expectancy and CVD mortality. Despite these mixed findings, our findings advocate for investing in more high-quality greenspaces in Latin American cities, given their scarcity in the region and their well-documented health benefits. However, future greening policies should make concerted efforts to promote health for all and apply a context-sensitive approach accounting for local social, economic and cultural characteristics to ensure that greenspaces do not further exacerbate existing health inequalities.

## Author contributions

MM, DR and UB conceived the study and developed the hypotheses and analysis plan. MM led the manuscript development and writing. UB conducted the statistical analyses and wrote substantial parts of the manuscript, including the methods section as well as the tables and figures. DR, UB, ID, YJ, KM, JJM, WTC and AALF participated in or supported data collection. JJM, NG, WTC, ID, YJ, KM and AALF provided feedback on the research design and the interpretation of results. UB and JJM contributed substantially to several versions of the writing of the manuscript. All authors read and approved the final version of the manuscript. DR supervised the research.

## Funding

The Salud Urbana en América Latina (SALURBAL)/Urban Health in Latin America project is funded by the 10.13039/100010269Wellcome Trust [205177/Z/16/Z]. UB was supported by the 10.13039/100000052Office of the Director of the National Institutes of Health under award number DP5OD26429. The funding sources had no role in the analysis, writing or decision to submit the manuscript.

## Declaration of competing interest

None declared.

## Figures and Tables

**Appendix 1:** cities' characteristics

city	n	ndvi	edu	pop	pop_growth	built_up	climate
Buenos Aires (AR)	51	0.51 [0.3;0.75]	-0.76 [-1.43;1.41]	15.13	4.40%	20.40%	Temperate
Belo Horizonte (BR)	21	0.77 [0.75;0.79]	-1.34 [-1.68;-0.89]	4.88	3.30%	13.70%	Tropical
Campinas (BR)	15	0.74 [0.72;0.75]	-0.2 [-0.75;0.7]	2.97	5.60%	17.50%	Tropical
Curitiba (BR)	11	0.85 [0.83;0.88]	-1.24 [-1.5;-0.51]	2.98	4.90%	11.70%	Temperate
Fortaleza (BR)	10	0.78 [0.73;0.84]	-1.57 [-2.14;-1.38]	3.54	3.20%	10.80%	Tropical
Porto Alegre (BR)	23	0.79 [0.74;0.83]	-1.32 [-1.75;-0.73]	3.7	2.40%	12.90%	Temperate
Recife (BR)	10	0.81 [0.79;0.84]	-1.24 [-1.46;-0.34]	3.64	2.50%	18.60%	Tropical
Rio de Janeiro (BR)	17	0.78 [0.71;0.83]	-1.04 [-1.42;-0.45]	12.09	2.10%	25.60%	Tropical
Sao Paulo (BR)	31	0.77 [0.52;0.83]	-0.38 [-1.09;0.14]	20.24	3.40%	32.70%	Temperate
Concepcion (CL)	10	0.87 [0.85;0.87]	-0.98 [-1.54;-0.28]	0.99	2.60%	6%	Temperate
Santiago (CL)	36	0.3 [0.24;0.48]	-1.1 [-1.42;0.01]	6.28	3.50%	27.30%	Arid
Bogota (CO)	15	0.78 [0.76;0.82]	-0.57 [-1.28;-0.12]	8.44	4.10%	13.60%	Temperate
San Jose (CR)	29	0.84 [0.72;0.87]	1.63 [0.31;2.66]	2.42	4.60%	11.10%	Tropical
Mexico City (MX)	76	0.64 [0.53;0.74]	-1.15 [-1.95;0.09]	20.81	2.90%	23.80%	Temperate
Monterrey (MX)	14	0.58 [0.51;0.66]	-1.04 [-2.09;-0.12]	4.37	6.90%	9.60%	Arid
Oaxaca de Juarez (MX)	22	0.65 [0.61;0.72]	-0.07 [-0.87;1.23]	0.63	4.60%	17.30%	Temperate
Orizaba (MX)	12	0.85 [0.84;0.86]	-1.18 [-2.04;-0.4]	0.44	5.40%	8.30%	Temperate
Puebla de Zaragoza (MX)	40	0.72 [0.68;0.75]	-1.58 [-2.42;-1.1]	2.99	6.20%	18.20%	Temperate
Tlaxcala (MX)	19	0.67 [0.66;0.69]	-0.99 [-1.48;-0.13]	0.52	6.80%	12.10%	Temperate
Toluca (MX)	16	0.77 [0.74;0.78]	-1.52 [-1.88;-1.21]	2.12	5.70%	13.10%	Temperate
Colon (PA)	11	0.9 [0.87;0.91]	0.34 [-0.87;1.18]	0.22	6.80%	2.90%	Tropical
David (PA)	18	0.86 [0.85;0.86]	-0.18 [-0.46;1.05]	0.21	3.90%	4.80%	Tropical
Panama City (PA)	53	0.81 [0.46;0.88]	0.26 [-0.55;1.8]	1.82	8.30%	7%	Tropical
Arequipa (PE)	19	0.32 [0.25;0.35]	2.27 [1.35;2.9]	0.89	4.50%	3.10%	Arid
Huancayo (PE)	11	0.67 [0.61;0.68]	0.52 [-0.09;0.88]	0.46	2.20%	7%	Temperate
Ica (PE)	10	0.18 [0.08;0.41]	1.12 [0.86;1.52]	0.32	4.90%	0.70%	Arid
Lima (PE)	51	0.19 [0.15;0.24]	1.77 [1.31;3.64]	9.47	6.40%	19.40%	Arid
San Salvador (SV)	20	0.85 [0.82;0.87]	-1.81 [-2.34;-0.56]	1.8	6.20%	16%	Tropical

AR = Argentina, BR = Brazil, CL = Chile, CO = Colombia, ES = El Salvador, MX = Mexico, PA = Panama, PE = Peru.

+: NDVI.

++: education index (z-score).

*: millions.

**: relative population growth over the 5 years of the study.

**Appendix 2:** Variables included in the analysis

	Definition	Units/Values (potential range)	Data source	Years
Geographical units				
Sub-city (L2)	Administrative units (e.g., municipios) nested within the city's administrative boundaries (L1Admin).	n/a	Political or administrative boundaries	Varies by sub-cities
City (L1 Admin)	A combination of adjacent administrative units (e.g., several municipios) that are part of the urban area/extent as determined from satellite imagery. This study included L1Admin units that include >10 L2 units.	n/a	Political or administrative boundaries	Varies by cities
Variables				
Sub-city variables				
Life expectancy	Expected number of years someone born today is expected to live if current mortality patterns hold in the future	Years of life (higher = longer lives)	Vital registration and population projections/estimations	2012–2016*
CMNN mortality	Age-adjusted mortality -adjusted mortality due to communicable, maternal, neonatal and nutritional conditions	Deaths/100,000 pop (higher = higher mortality)	Vital registration and population projections/estimations	2012–2016*
CVD mortality	Age-adjusted mortality due to cardiovascular diseases	Deaths/100,000 pop (higher = higher mortality)	Vital registration and population projections/estimations	2012–2016*
Violence-related mortality	Age-adjusted mortality due to suicides or homicides	Deaths/100,000 pop (higher = higher mortality)	Vital registration and population projections/estimations	2012–2016*
Greenness	Summary statistics (median) of annual maximum NDVI values in a geographical unit.	Ranging from −1 to 1 (no units), with higher values indicate more vegetated areas that are also greener	MODIS product	2012–2016
High school education	Proportion of the population aged 25 or older who completed secondary education (i.e., 12 years of school education) or above	Ranges from 0 to 100.	Census data of each country	Varies by country: Argentina (2010), Brazil (2010), Chile (2002), Colombia (2005), Mexico (2010), Panama (2010) and El Salvador (2007)
College education or higher	Proportion of the population aged 25 or older who completed university education (i.e., 4 years of university education) or above	Ranges from 0 to 100.	Census data of each country	
% population aged 0-15	Proportion of the population aged 0-15	Ranges from 0 to 100.	Census data of each country	2012–2016
% population aged 65+	Proportion of the population aged 65+	Ranges from 0 to 100.	Census data of each country	2012–2016
Distance to city center	The airline distance between the center of each sub-city units to city center.	Kilometers, with higher values indicating longer distance to the city center.	Geospatial location of L2 units - centroids	n/a
City level variables				
Greenness (city level)	Summary statistics (median) of annual maximum NDVI values in a geographical unit.	Ranging from −1 to 1 (no units), with higher values indicating more vegetated areas that are also greener	MODIS product	2012–2016
Total population	Total population residing within the city's administrative boundary.	Higher values indicate more people residing in the unit.	World pop***	2010
Population growth	Relative change in population in the last 5 years	% change (higher = higher growth)	Population projections/estimations	2012–2016
% built-up	Total urban area divided by the total area and multiplied by 100.	Ranges from 0 to 100, with higher values indicating higher urban development.	The Global urban footprint project**	2012
Climate zone group	Major climate zone in a geography, indicating its seasonal precipitation and temperature patterns.	Level-1 categories (tropical, temperate, continental, arid and polar climates)	Köppen climate classification	n/a

*Salvador's mortality data was for 2010–2014 due to restricted data availability.

**the Global Urban Footprint project: https://www.dlr.de/eoc/en/desktopdefault.aspx/tabid-9628/16557_read-40454/.

***World pop: https://www.worldpop.org/.

****Köppen climate classification.

**Appendix 3a:** Association between area-level education and life expectancy and mortality by levels of greenness

		Life expectancy	CMNN mortality		CVD mortality		Violence related mortality	
		β, 95% CI∗	RR, 95% CI^	Relative change§	RR, 95% CI^	Relative change§	RR, 95% CI^	Relative change§
Women								
-2SD greenness	(NDVI = 0·24)	0·05 (−0·13; 0·23)	0·98 (0·96; 1·01)	−2%	0·99 (0·97; 1·01)	−1%	**0·95 (0·91; 0·99)**	**−5%**
-1SD greenness	(NDVI = 0·47)	**0·19 (0·08; 0·31)**	**0·97 (0·96; 0·99)**	**−3%**	**0·97 (0·96; 0·99)**	**−3%**	**0·96 (0·93; 0·98)**	**−4%**
Mean greenness	(NDVI = 0·70)	**0·34 (0·23; 0·45)**	**0·96 (0·94; 0·97)**	**−4%**	**0·96 (0·95; 0·97)**	**−4%**	**0·96 (0·93; 0·99)**	**−4%**
+1SD greenness	(NDVI = 0·93)	**0·49 (0·32; 0·66)**	**0·95 (0·92; 0·97)**	**−5%**	**0·94 (0·93; 0·96)**	**−6%**	0·97 (0·93; 1·01)	−3%
								
Men								
-2SD greenness	(NDVI = 0·24)	0·10 (−0·14; 0·33)	0·98 (0·95; 1·00)	−2%	0·99 (0·97; 1·01)	−1%	**0·84 (0·80; 0·88)**	**−16%**
-1SD greenness	(NDVI = 0·47)	**0·30 (0·15; 0·45)**	**0·97 (0·95; 0·98)**	**−3%**	**0·98 (0·97; 0·99)**	**−2%**	**0·87 (0·84; 0·89)**	**−13%**
Mean greenness	(NDVI = 0·70)	**0·51 (0·36; 0·65)**	**0·96 (0·94; 0·97)**	**−4%**	**0·96 (0·95; 0·98)**	**−4%**	**0·90 (0·87; 0·92)**	**−10%**
+1SD greenness	(NDVI = 0·93)	**0·71 (0·49; 0·93)**	**0·95 (0·92; 0·97)**	**−5%**	**0·95 (0·93; 0·97)**	**−5%**	**0·92 (0·89; 0·96)**	**−8%**

Footnote.

∗ Results for life expectancy are beta coefficients (95% CI), interpreted as the increase in years of life expectancy per 1 SD increase in education at −2, −1, 0, and +1 SD of greenness (as presented in Fig. 1).

^ Results for cause-specific mortality are Rate Ratios (95% CI), interpreted as the relative change in CVD/CMNN/Violence-related mortality per 1 SD increase in education at −2, −1, 0, and +1 SD of greenness (as presented in Fig. 1).

§ Relative change in mortality per 1 SD increase in education at −2, −1, 0, and +1 SD of greenness. Coefficients with a p-value <0·05 are in bold.

**Appendix 3b:** Association between greenness and life expectancy and mortality by different levels of area-level education

	Life expectancy	CMNN mortality			CVD mortality		Violence related mortality	
	β, 95% CI∗∗	RR, 95% CI^	Relative change§		RR, 95% CI^	Relative change§	RR, 95% CI^	Relative change§
Women∗								
-2SD education	−0·33 (−0·68; 0·01)	0·99 (0·94; 1·03)		−1%	1·04 (1·01; 1·08)	+4%	0·98 (0·90; 1·06)	−2%
-1SD education	−0·19 (−0·49; 0·12)	0·97 (0·93; 1·01)		−3%	1·03 (1·00; 1·06)	+3%	0·98 (0·91; 1·06)	−2%
Mean education	−0·04 (−0·33; 0·24)	0·96 (0·92; 1·00)		−4%	1·01 (0·98; 1·04)	+1%	0·99 (0·92; 1·06)	−1%
+1SD education	0·11 (−0·19; 0·4)	**0·95 (0·91; 0·99)**		**−5%**	1·00 (0·97; 1·03)	0%	0·99 (0·92; 1·07)	−1%
							
Men∗								
-2SD education	−0·02 (−0·46; 0·43)	0·97 (0·92; 1·02)		−3%	1·02 (0·98; 1·06)	+2%	**0·87 (0·80; 0·95)**	**−13%**
-1SD education	0·19 (−0·2; 0·57)	0·96 (0·92; 1·00)		−4%	1·00 (0·97; 1·04)	0%	**0·90 (0·84; 0·97)**	**−10%**
Mean education	**0·39 (0·03; 0·75)**	**0·95 (0·91; 0·99)**		**−5%**	0·99 (0·96; 1·02)	−1%	0·93 (0·87; 1·00)	−7%
+1SD education	**0·6 (0·22; 0·97)**	**0·94 (0·9; 0·98)**		**−6%**	0·98 (0·95; 1·01)	−2%	0·96 (0·90; 1·03)	−4%

∗ we do not provide raw education %s corresponding to standard deviation levels of the area-level education index, since this is a composite index with two indicators (% high school and % university education.

∗∗ Results for life expectancy are beta coefficients (95% CI), interpreted as the increase in years of life expectancy per 1 SD increase in education at −2, −1, 0, and +1 SD of greenness (as presented in Fig. 1).

^ Results for cause-specific mortality are Rate Ratios (95% CI), interpreted as the relative change in CVD/CMNN/Violence-related mortality per 1 SD increase in education at −2, −1, 0, and +1 SD of greenness (as presented in Fig. 1).

§ Relative change in mortality per 1 SD increase in education at −2, −1, 0, and +1 SD of greenness. Coefficients with a p-value <0·05 are in bold.
